# Phosphatidylinositol-4,5-Bisphosphate Enhances Anionic Lipid Demixing by the C2 Domain of PKCα

**DOI:** 10.1371/journal.pone.0095973

**Published:** 2014-04-24

**Authors:** Antonio L. Egea-Jiménez, Ana M. Fernández-Martínez, Ángel Pérez-Lara, Ana de Godos, Senena Corbalán-García, Juan C. Gómez-Fernández

**Affiliations:** Departamento de Bioquímica y Biología Molecular-A, Facultad de Veterinaria, Regional Campus of International Excellence “Campus Mare Nostrum”, Universidad de Murcia, Murcia, Spain; University of Pittsburgh School of Medicine, United States of America

## Abstract

The C2 domain of PKCα (C2α) induces fluorescence self-quenching of NBD-PS in the presence of Ca^2+^, which is interpreted as the demixing of phosphatidylserine from a mixture of this phospholipid with phosphatidylcholine. Self-quenching of NBD-PS was considerably increased when phosphatidylinositol-4,5-bisphosphate (PIP_2_) was present in the membrane. When PIP_2_ was the labeled phospholipid, in the form of TopFluor-PIP_2_, fluorescence self-quenching induced by the C2 domain was also observed, but this was dependent on the presence of phosphatidylserine. An independent indication of the phospholipid demixing effect given by the C2α domain was obtained by using ^2^H-NMR, since a shift of the transition temperature of deuterated phosphatidylcholine was observed as a consequence of the addition of the C2α domain, but only in the presence of PIP_2_. The demixing induced by the C2α domain may have a physiological significance since it means that the binding of PKCα to membranes is accompanied by the formation of domains enriched in activating lipids, like phosphatidylserine and PIP_2_. The formation of these domains may enhance the activation of the enzyme when it binds to membranes containing phosphatidylserine and PIP_2_.

## Introduction

Protein kinase C (PKC) is a family of related protein kinases that participate in cell control. The PKCs family includes eleven different mammalian isoenzymes, which are usually subdivided into three groups. Those in the first group are called classical or conventional (α, βI, βII and γ) and they are activated by anionic phospholipids, diacylglycerol (or phorbol esters) and by Ca^2+^. The second group includes the isozymes called novel or new (δ, ε, η and θ), which are also regulated by anionic phospholipids and diacylglycerols (or phorbol esters) but not by calcium. Finally there is another group which is relatively different, which includes the isoenzymes called atypical (ζ and ι/λ), which are not regulated neither by diacylglycerol nor by calcium [1]–[3]. See for comprehensive reviews about this family of enzymes the following references [4]–[7].

Classical PKCs are activated, at least, at three different sites; by diacylglycerols interacting with the C1 domain [6], by Ca^2+^ acting at the C2 domain, which bridges with membrane phospholipids, especially phosphatidylserine [8]–[10] and by PIP_2_, which also acts at the C2 domain [11], [12]. In addition, membrane effects arising from different molecules like diacylglycerols may also activate these enzymes [13]. Interestingly, it has been shown that the site to where PIP_2_ (phosphatidylinositol-4,5-bisphosphate) binds in the C2 domain of classical PKCs is a groove formed by strands β3 and β4, which is rich in lysine residues and is sometimes called the polylysine site and the three-dimensional structure of the domain bound to soluble PIP_2_ was determined in pioneering studies [11], [12], [14]. This site has been shown to exhibit high specificity for PIP_2_ [12], [15]–[23] although it may also bind other lipids like phosphatidic acid or phosphatidylserine [11] or even retinoic acid [24].

It has been observed that negatively charged lipids are recruited to some protein binding sites as a result of their affinity for the protein [25]. It has been shown recently that another C2 domain, like that of synaptotagmin 1 [26], is able to induce demixing of phosphatidylserine and the aggregation of different membranes, such aggregation being attributed to the combined effect of C2A and C2B domains.

We used fluorescent probes in this work to show that the C2 domain of PKCα may induce the demixing of POPS (1-palmitoyl-2-oleoyl-*sn*-phosphatidylserine) in a membrane in which POPC (1-palmitoyl-2-oleoyl-*sn*-phosphatidylcholine) was also present, an effect that is Ca^2+^-dependent. Surprisingly, the demixing of phosphatidylserine by the C2 domain was very much increased if PIP_2_ was also present. Similarly, the C2 domain may also induce the demixing of PIP_2_ from (POPC) when Ca^2+^ is present but this effect requires the presence of POPS. In addition ^2^H-NMR showed that POPC is demixed from a POPC/POPS/PIP_2_ membrane in the presence of Ca^2+^ and no demixing was detected in the absence of PIP_2_.

## Experimental

### Materials

1-Palmitoyl-2-oleoyl-*sn*-glycero-3-phosphoserine (POPS); 1-palmitoyl (d_31_)-2-oleoyl-*sn*-glycero-3-phosphoserine (POPS-d_31_); 1-palmitoyl-2-oleoyl-*sn*-glycero-3-phosphocholine (POPC); 1-palmitoyl (d_31_)-2-oleoyl-sn- glycero-3-phosphocholine (POPC-d_31_), phosphatidylinositol 4,5-bisphosphate (PIP_2_); 1-oleoyl-2-[6-[(7-nitro-2-1,3-benzoxadiazol-4-yl) amino] hexanoyl]-*sn*-glycero-3-phosphoserine (NBD-PS); 1-oleoyl-2-[6-[4-(dipyrrometheneborondifluoride)butanoyl]amino]hexanoyl-*sn*-glycero-3-phosphoinositol-4,5-bisphosphate (TopFluor- PIP_2_, a derivative of BODIPY) were purchased from Avanti Polar Lipids (Birmingham, LA, U.S.A.). All other chemicals were highly pure and were obtained from Sigma Chemical Co. (Madrid, Spain).

### Construction of Expression Plasmids

PKCα cDNA was a kind gift from Drs. Nishizuka and Ono (Kobe University, Kobe, Japan).

The cDNA encoding the C2 domain of PKCα (residues 158–285) was amplified by PCR using the following primers:

C2α:5′ CAAGAATTCAAGAGGGGGCGGATTTAC

C2α:3′ CAAAAGCTTGTATTCACCCTCCTCTTG

The resulting 381 bp PCR fragment was subcloned into the HindIII and EcoRI sites of the bacterial expression vector pET28c(+), in which the insert is fused to a six-histidine tag. The construct was confirmed by DNA sequencing.

### Expression and purification of the His-PKCα-C2 domain

Expression and purification of C2 domains were performed essentially as described in [27]. Briefly, the pET28c(+) plasmid containing PKC-C2 domains were transformed into BL21 (DE3) Escherichia coli cells. Once the bacterial cultures had reached an OD 600 of approximately 0.6, they were induced by adding 0.5 mM isopropyl 1-thio-β-D-galactopyranoside (IPTG) for 5 h at 30°C. The cells were resuspended in lysis buffer containing 25 mM Hepes pH 7.4 and 300 mM NaCl and protease inhibitors (10 mM benzamidine, 1 mM PMSF, and 10 µg/mL-1 trypsin inhibitor) and lysed by sonication on ice for 10 seconds 10 times. The clarified supernatants were incubated with Ni-NTA agarose for 2 h at 4°C. The Ni beads were then washed with 20 ml of lysis buffer containing 20 mM imidazole, and the bound proteins were eluted by applying the same buffer containing 50, 250, and 500 mM imidazole. The sixhistidine tag was removed by thrombin digestion, finally, the C2α domain was washed with the same buffer and concentrated using an Ultrafree-5 centrifugal filter unit (Millipore Inc, Bedford, MA).

Protein concentrations were determined at 280 nm [28]. The purity of the samples was checked by a SDS-15% (w/v) polyacrylamide gel electrophoresis [29] and Coomassie blue (Sigma, St. Louis, MO, USA) staining, which revealed that it was higher than 95%. The native folding of the recombinant C2 domain produced in our laboratory was widely confirmed by our previous X ray crystallographic studies with the PKCα-C2 domain [11], [14], [24] and also by FTIR spectroscopy [30], [31].

### Preparation of lipid vesicles

Lipid vesicles for FRET experiments were generated by mixing chloroform lipid solutions in the desired proportions and then dried from the organic solvent under a stream of oxygen-free nitrogen. The last traces of organic solvent were removed under vacuum for at least 3 h. Dried phospholipids mixtures were resuspended in the corresponding buffers by vigorous vortexing. Large unilamellar phosholipid vesicles (LUV) (100 nm in diameter) were generating by extruding the lipid emulsion through two stacked polycarbonate membranes (Millipore Inc., Bedford, USA) with a pore size of 100 nm (11 passes).

Samples for NMR were prepared using a total quantity of 15 mg of phospholipid, dissolving the desired amounts of phospholipids in chloroform before being mixed. Samples were then dried under a nitrogen stream and finally under vacuum. When the sample consisted of lipid only, this mixture was dispersed in 100 mM NaCl, 25 mM Hepes pH 7.4 buffer, 1 mM CaCl_2_ using deuterium-depleted water. If the sample also included protein, a sufficient amount of lyophilized C2 domain protein to give the final lipid/protein molar ratio of 40∶1 was dissolved in the buffer prepared with water, or with deuterium-depleted water in the case of ^2^H-NMR, and added to the dried lipid to prepare multilamellar vesicles. The sample was then centrifuged to eliminate unbound protein and the pellet dispersed in 300 µl of buffer and transferred to NMR glass tubes.

### Fluorescence measurements

Steady-state fluorescence measurements were carried out on a Fluoromax-3 fluorimeter (Jobin Yvon, Edison, NJ) at 25°C in an assay buffer composed of 25 mM Hepes pH 7.4, 100 mM NaCl and 250 µM CaCl2. All measurements were performed at 25°C. Fluorescence intensities were determined according to Lakowicz [32]. For each measurement a total volume of 1.5 ml was used and desired amounts of protein were titrated into the LUV suspension and incubated for 10 min before each measurement. NBD-PS emission spectra was obtained using a 50 µM lipid suspension, by scanning from 490 nm to 600 nm with the excitation wavelength set to 460 nm (4 nm excitation and 4 nm emission slit widths). TopFluor-PIP_2_ emission spectra was obtained using a 50 µM lipid suspension by scanning from 498 nm to 600 nm with the excitation wavelength set to 490 nm (4 nm excitation and 4 nm emission slit widths).The fluorescence decreases due to the change in volume produced by the addition of protein solution were corrected by subtracting the value obtained by adding the same volume of buffer without protein as a blank. The percent quenching was calculated according with the equation: 
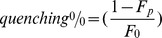
where *F_p_* and *F_0_* are the fluorescence intensity in the presence or in the absence of protein respectively.

### ^2^H –NMR experiments

^2^H-NMR experiments were carried out on a Bruker Avance 600 instrument (Bruker, Etlingen, Germany) at 92.123 MHz using the standard quadrupole echo sequence [33]. The spectral width was 150 KHz, with a 10 µs 90° pulse, 40 µs pulse spacing, 3.35 µs dwell time, 1 s recycling time and 50 Hz line broadening, with an accumulation of 15000 transients. Spectra were acquired at temperatures ranging from −14°C to 22°C, raising the temperature in 2°C steps. The first moment, *M_1_*, was calculated for each spectrum of the different samples and at each temperature using the following equation [34]. 
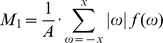
where ω is the frequency shift from the central (Larmor) frequency, *f(ω)* is the spectral intensity, *x* is the frequency shift range (between −60 and 60 kHz) and A is defined as:
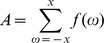



## Results

### PKCα C2 domain induces demixing of POPS as seen by fluorescence quenching

**Figure 1:**
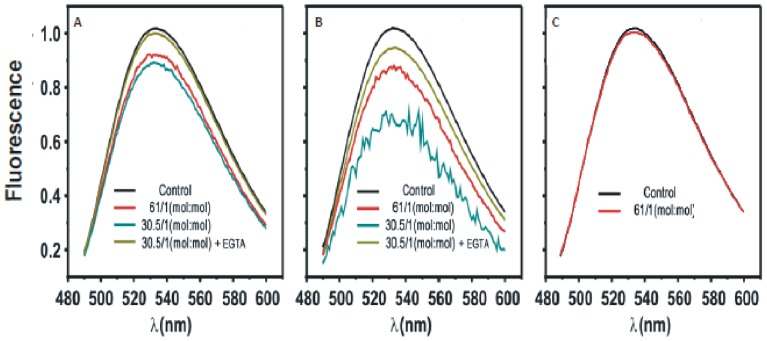
Fluorescence spectra of NBD-PS in LUVs in the presence of different molar ratios of total lipid to protein (C2α domain) at different lipid compositions. The molar ratio of total lipid to protein are indicated. (A) POPC/POPS/NBD-PS (molar ratio 80/19.5/0.5), (B) POPC/POPS/PIP2/NBD-PS in molar ratio 75∶19.5∶5∶0.5. (C) POPC/NBD-PC (molar ratio 99.5/0.5). A Ca^2+^ concentration of 100 µM was present in the assays, but control samples to which 3 mM EGTA was added instead of Ca^2+^ are shown.

**Figure 2:**
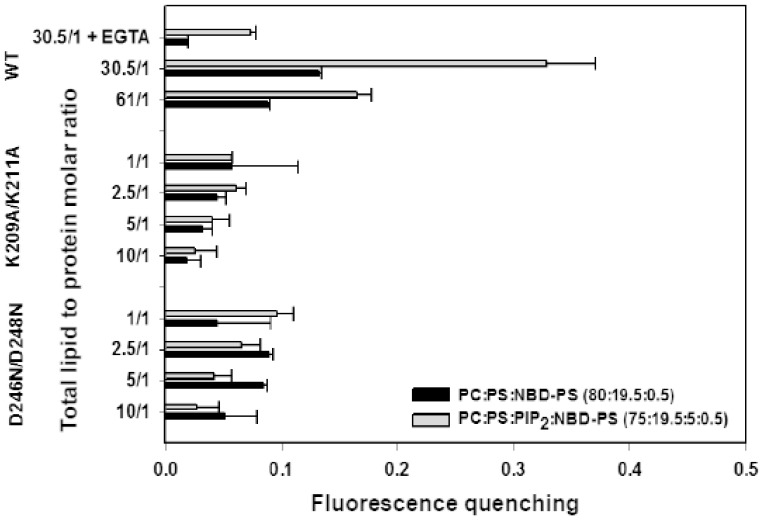
Fractional fluorescence quenching of NBD-PS as a function of the molar ratio of total lipid to protein (C2α domain) and the indicated mutated forms of the domain as shown in each case. A Ca^2+^ concentration of 250 mM was present in the assays, but a control sample to which 3 mM EGTA was added instead of Ca^2+^ is shown. Error bars indicate the S.E. (n = 3).

Figure 1 shows that C2α induces a moderate quenching of the fluorescence of POPS labeled with NBD in one of their fatty-acyl chains. NBD-PS is used as a fluorescent analogue of POPS and it has been reported to be a self- quenching fluorophore [26]. The extent of self-quenching depends on the distance between fluorophores and it will increase if POPS molecules are demixed due to the action of the C2 domain. In a first assay LUV membranes were composed of POPC/POPS/NBD-PS in molar ratio 80∶19.5∶0.5 (Fig. 1A). Increasing concentrations of the C2 domain gave place to increasing fluorescence quenching (Fig. 1A), although as seen in Fig. 2, maximum quenching is about 13% at the maximum lipid to protein ratio tested. On the other hand, addition of EGTA produced a recovery of the fluorescence indicating that the effect depends on the presence of Ca^2+^ so that in its absence the level of quenching is about 3% only, approaching the experimental error (Fig. 2). The observed quenching is attributed to self-quenching due to aggregation of POPS as a consequence of the interaction with the C2 domain of PKCα.

A clearly bigger effect of the C2 domain was observed when PIP_2_ was incorporated in the membrane (Fig. 1B) so that the concentration was POPC/POPS/PIP_2_/NBD-PS in molar ratio 75∶19.5∶5∶0.5. It can be observed that the spectra obtained for this last sample (Fig, 1B) were noisier than those in the absence of PIP_2_ (Fig. 1A) and this is probably due to aggregation of the vesicles. Nevertheless this aggregation did not affect substantially to the result, since a control experiment in which water soluble NBD-glycine was added to POPC/POPS/PIP_2_ (non-fluorescent) vesicles showed that there was a small increase in apparent fluorescence, due to scattering, which was lower of 5% of the signal even with the maximum concentration of C2 (results not shown).

As it can be seen in Fig. 2, in the presence of PIP_2_ a higher quenching effect was obtained, going in this case up to 32% at the maximum lipid to protein molar ratio tested (30.5∶1, total lipid to protein) and this is interpreted as if PIP_2_ would facilitate aggregation of NBD-PS induced by C2 domain. It can be observed that this effect also depends on Ca^2+^ and the addition of EGTA produced a recovery of fluorescence, although a residual quenching of about 8% remains in the absence of Ca^2+^ (Fig. 2) probably because the interaction of PIP_2_ with the C2α domain is not totally dependent on Ca^2+^ [18]. Fig. 1C shows a control experiment in which C2α domain was added to a POPC membrane containing 0.5 mol% of PC-NBD. It can be observed that no quenching effect was observed in this case in which anionic lipids are not present.

**Figure 3:**
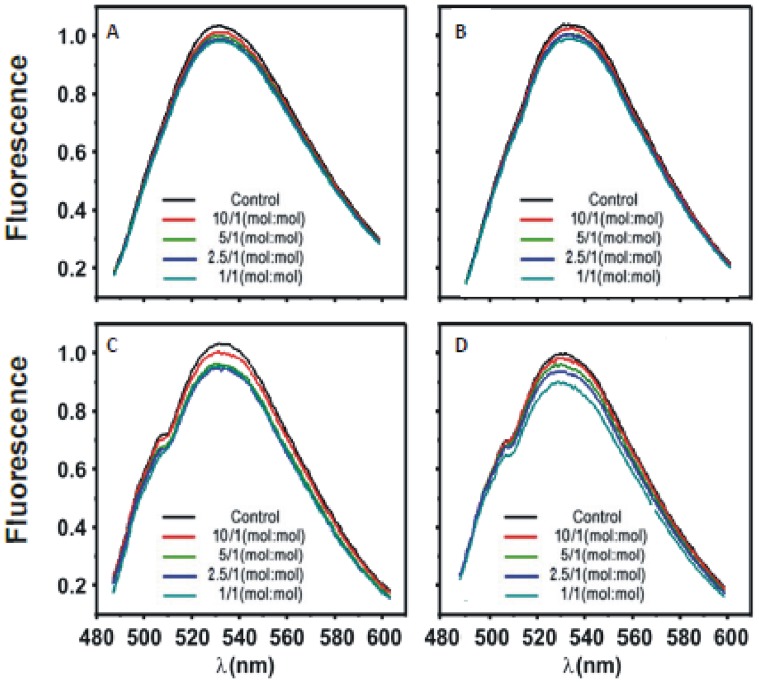
Fluorescence spectra of NBD-PS in LUVs in the presence of different molar ratios of total lipid to protein concentrations (C2α domain) and some mutated forms, the molar ratios of total lipid to protein are indicated. (A) POPC/POPS/NBD-PS (molar ratio 80/19.5/0.5) in the presence of C2α D246N/D248N, (B) POPC/POPS/PIP_2_/NBD-PS in molar ratio 75∶19.5∶5∶0.5 in the presence of C2α D246N/D248N. (C) POPC/POPS/NBD-PS (molar ratio 80/19.5/0.5) in the presence of C2α K209A/K211A, (D) POPC/POPS/PIP_2_/NBD-PS in molar ratio 75∶19.5∶5∶0.5 in the presence of C2α. A Ca^2+^ concentration of 100 µM was present in the assays.

Fig. 3A shows that if site-directed mutations were introduced in the calcium binding site (D246/248N) [14] the addition of C2α domain to membranes containing POPC/POPS/NBD-PS (79∶20∶0.5, molar ratio) did not produce any substantial self-quenching effect with a maximum observed quenching of about 4% when the total molar lipid/protein was increased to 1∶1 (Figs. 2 and 3). The same lack of substantial effect was observed when a membrane containing POPC/POPS/PIP_2_/NBD-PS (74.5∶20∶5∶0.5, molar ratio) was used (Fig. 3B), with a maximum quenching of about 9% when the molar total lipid to protein ratio was 1∶1 (Fig. 2). This shows that the calcium binding site of the C2α domain is essential to induce lipid demixing.

Similar results were observed when the site-directed mutation (Fig. 3C) was introduced onto the PIP_2_–binding site, i.e. K209A/K211A, which has been shown to form part of this site [12]. When this modified C2α domain was added to POPC/POPS/NBD-PS (80∶20∶0.5 molar ratio) (Fig. 3C) the maximum observed quenching was about 6% when the molar total lipid to protein was 1∶1 (see Fig. 2) and with POPC/POPS/PIP_2_/NBD-PS (75∶20∶5∶0.5 molar ratio) (Fig. 3D) membranes, the self-quenching effect was very much reduced to about 7% (Fig. 2). These results underline the lack of capacity of the mutated protein to induce this effect when either of the two lipid binding sites of the protein was disabled.

**Figure 4:**
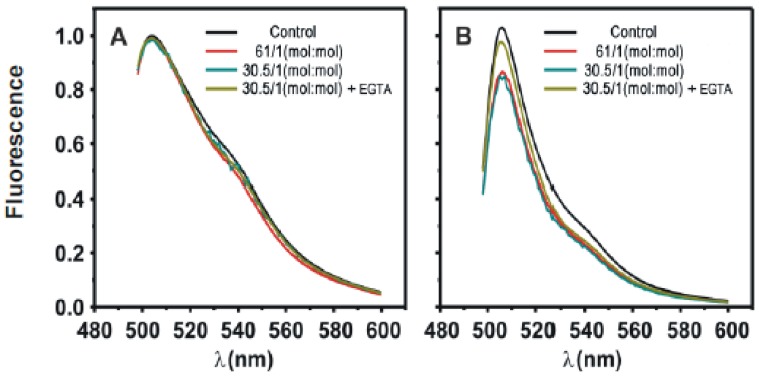
Fluorescence spectra of TopFluor-PIP_2_ in LUVs containing POPC/PIP_2_/TopFluor- PIP_2_ in molar ratio 95∶4.5∶0.5 (A) and POPC/POPS/PIP_2_/TopFluor- PIP_2_ in molar ratio 75∶20∶4.5∶0.5 (B) with different molar ratios of total lipid to protein (C2α domain) as indicated. A Ca^2+^ concentration of 250 µM was present in the assays, but control samples to which 3 mM EGTA was added instead of Ca^2+^ are shown.

**Figure 5:**
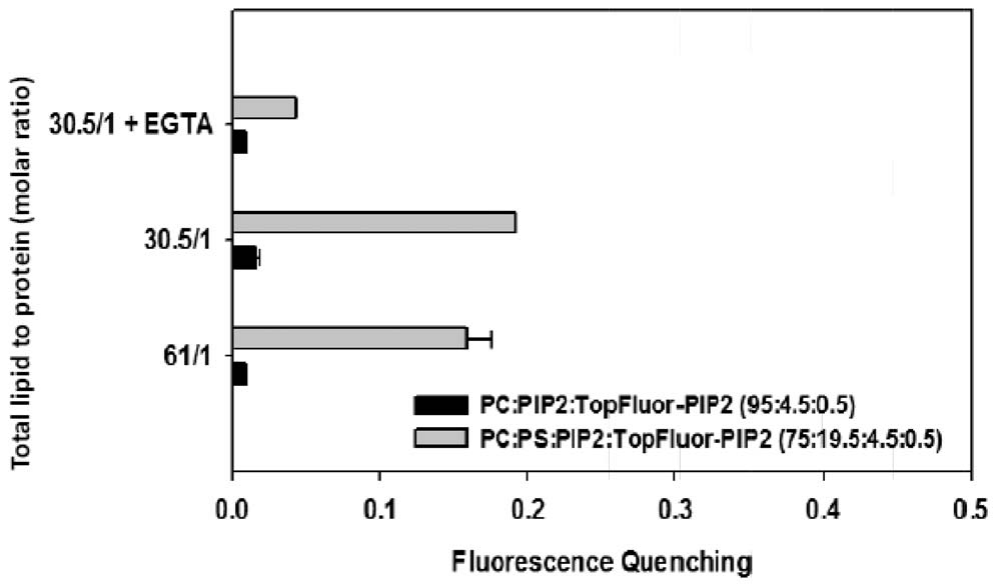
Fractional fluorescence quenching of PIP_2_-TopFluor as a function of the molar ratios of total lipid to protein. Molar ratios are indicated in each case. A Ca^2+^ concentration of 250 µM was present in the assays, but a control sample to which 3 mM EGTA was added instead of Ca^2+^ is shown. Error bars indicate the S.E. (n = 3).

In order to check if C2α domain may also induce the aggregation of PIP_2_, a fluorescent analogue as TopFluor-PIP_2_ was used, labelled in one of the fatty acyl chains. BODIPY fluorescent probes have been also found to suffer self-quenching in lipid vesicles [35]. If POPS was not present, with membranes composed of POPC/PIP_2_/TopFluor-PIP_2_ (95∶4.5∶0.5 molar ratio), Fig. 4A shows that the quenching was not occurring within experimental error when C2α was added (see also Fig. 5). When the lipid vesicles were composed of POPC/POPS/PIP_2_/TopFluor-PIP_2_ (75∶20∶5∶0.5 molar ratio) it was observed that some quenching occurred, being about 19.5% when the molar ratio of total lipid to protein was 30.5 (Fig. 4A and Fig. 5) and therefore it seems that the C2α domain is also able of demixing this phosphoinositide. This effect was also dependent on Ca^2+^ and the quenching decreased to about 1% in the presence of EGTA (see Fig. 5).

### ^2^H-NMR spectroscopy studies

**Figure 6:**
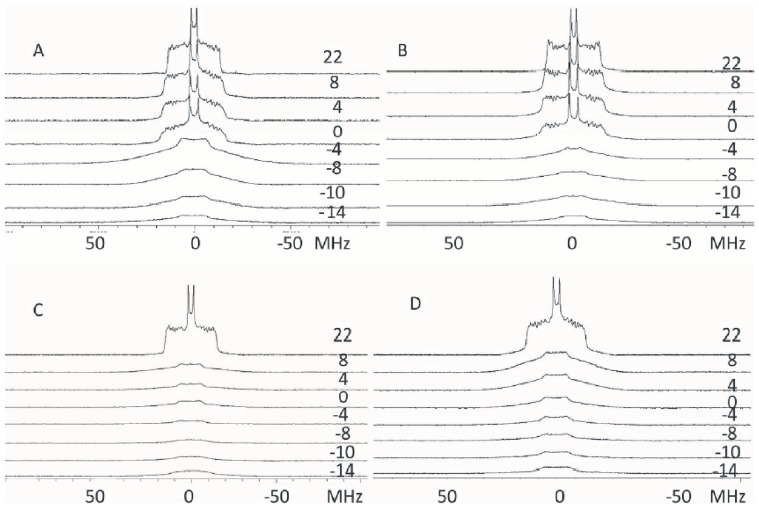
^2^H-NMR representative spectra obtained at the temperatures shown. (A) POPC/POPC-d_31_ (2.5∶1, molar ratio); (B) POPC/POPC-d_31_ (2.5∶1, molar ratio) in the presence of C2α domain (40∶1 phospholipid/protein molar ratio); (C) POPS/POPS d_31_ (3∶1 molar ratio) and (D) POPS/POPS d_31_ (3∶1 molar ratio) in the presence of C2α domain (40∶1 phospholipid/protein molar ratio).

**Figure 7:**
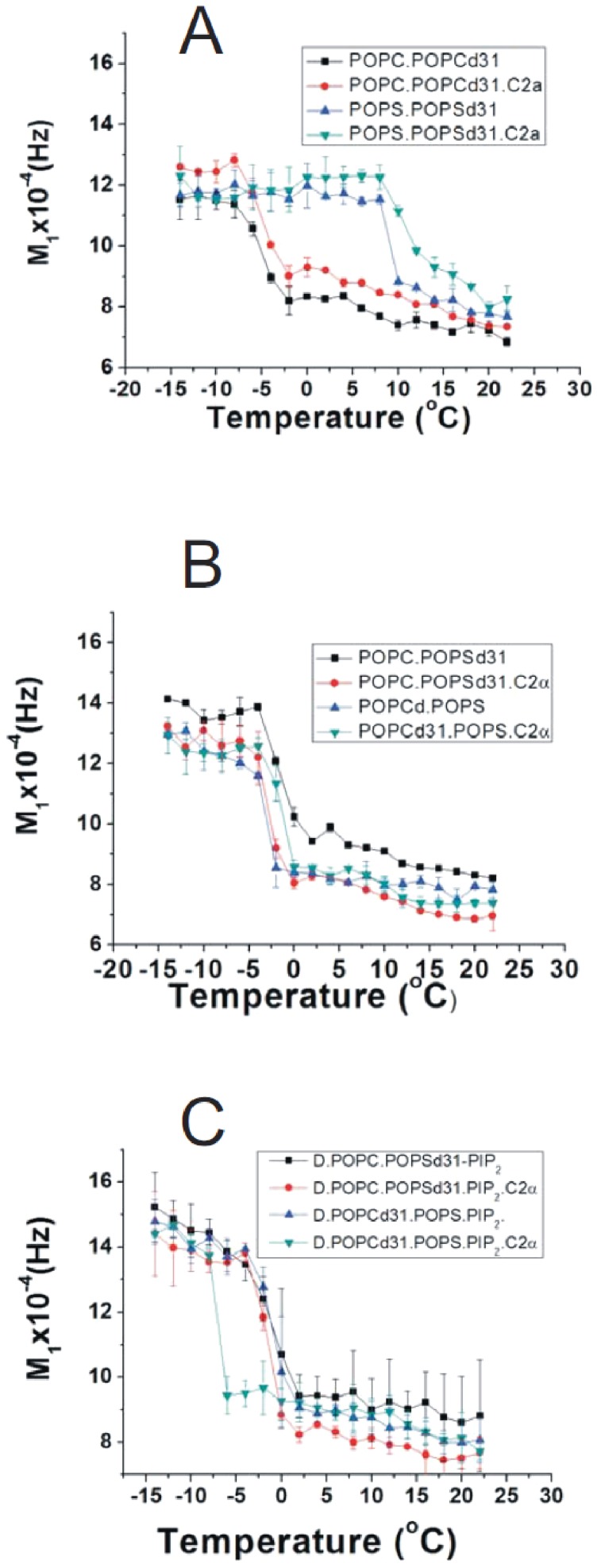
Temperature-dependent changes of the *M_1_* first moment versus temperature, for different samples as shown in each case. (A) POPC/POPC-d_31_ (2∶1, molar ratio); POPC/POPC-d_31_ (2∶1, molar ratio) in the presence of C2α domain (40∶1 phospholipid/protein molar ratio); POPS/POPS d_31_ (2∶1 molar ratio) and POPS/POPS d_31_ (2∶1 molar ratio) in the presence of C2α domain (40∶1 phospholipid/protein molar ratio). (B) POPC/POPC d_31_/POPS (40∶40∶20, molar ratio); POPC/POPC d_31_/POPS (40∶40∶20, molar ratio) in the presence of C2α (molar ratio phospholipid/protein 40∶1); POPC/POPS d_31_ (80∶20, molar ratio); POPC/POPS d_31_ (80∶20, molar ratio) in the presence of C2α (molar ratio phospholipid/protein 40∶1). (C) POPC/POPC-d_31_/POPS/PIP_2_ (35∶40∶20∶5, molar ratio); POPC/POPC-d_31_/POPS/PIP_2_ (35∶40∶20∶5, molar ratio); POPC/POPS-d_31_/PIP_2_ (75∶20∶5, molar ratio and POPC/POPS-d_31_/PIP_2_ (75∶20∶5, molar ratio) in the presence of C2α domain (40∶1 phospholipid/protein molar ratio). Error bars indicate the S.E. (n = 3).

To further confirm the phospholipid demixing indicated by the FRET experiments, ^2^H-NMR experiments were designed in the presence and in the absence of C2α domain. In some of these experiments deuterated POPC (POPC-d_31_) was used and in others the deuterated phospholipid was POPS (POPS-d_31_). Spectra were measured at different temperatures. When pure POPC, in a mixture POPC/POPCd_31_ (molar ratio 2.5∶1) was studied (Fig. 6A) at temperatures below the transition temperature (*T_c_*) of the pure phospholipid, the spectra were powder patterns characteristic of a gel phase. At higher temperatures (0°C and higher), the spectra corresponded to a fluid-phase bilayer and were axially symmetric with some resolved quadrupole splittings arising from methylene segments in the acyl chains. In order to better discern the phase transition and other effects of the C2α domain on these membranes, the first spectral moment *M_1_* was calculated for each spectrum. *M_1_* measures the average spectral width, and since each phase has a distinct spectral width it will also have a characteristic *M_1_*, which is proportional to the average order parameter. The variations in *M_1_* with temperature can be used to characterize membrane phase transitions and the molecular order of a membrane. The phase transition has its onset at −8°C and it ends at −2°C (Fig. 6A and Fig. 7). In this case, the addition of C2α domain did not have any effect on the phase transition (Fig. 6B and Fig. 7A).

When POPS was the labeled phospholipid (POPS/POPSd_31_, 3∶1 molar ratio), spectra that indicate gel state of the membrane were observed up to 8°C (Fig. 6C) and as shown by the *M_1_* dependence on temperature (Fig. 7A), the transition temperature of gel to fluid begins at 8°C and it is very cooperative being completed at 10°C. The addition of C2α to POPS broadens the transition (Fig. 6D) and this can be clearly observed by the variation in the spectral moment *M_1_* (Fig. 7A) and although the onset remains at 8°C it tails out until 20°C.

**Figure 8:**
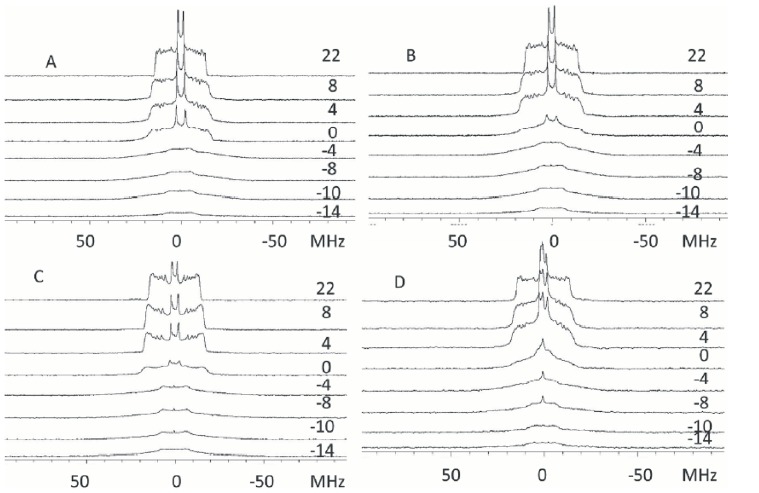
^2^H-NMR representative spectra obtained at the temperatures shown. (A) POPC/POPC d_31_/POPS (40∶40∶20, molar ratio); (B) POPC/POPC d_31_/POPS (40∶40∶20, molar ratio) in the presence of C2α (molar ratio phospholipid/protein 40∶1); (C) POPC/POPS d_31_ (80∶20, molar ratio) and (D) POPC/POPS d_31_ (80∶20, molar ratio) in the presence of C2α (molar ratio phospholipid/protein 40∶1).

In the next step the mixture POPC/POPCd_31_/POPS (40∶40∶20, molar ratio) was studied and a gel pattern of the spectra was observed until −4°C and a fluid pattern at higher temperatures (Fig. 8A). Fig. 7B shows a plot of *M_1_* versus temperature that indicates an onset of the phase transition at −4°C ending at −2°C. As seen in Fig. 8B the addition of C2α did not change the pattern of the spectra or the phase transition (Fig. 7B). If the labeled phospholipid was POPS (POPC/POPSd_31_, 80∶20 molar ratio) (Fig. 8C) the pattern of the spectra was similar to those seen above, when POPC was the labeled one in the absence of protein, demonstrating that before the addition of C2α there is a good mixing of the phospholipids and the onset of the phase transition is at −4°C, i.e. the same than it can be observed for the mixture in which the deuterated lipid is POPC, in the absence of protein (Fig. 7B). It should be noted, however, that when C2α was added to this mixture (POPC/POPSd_31_, 80∶20 molar ratio), the spectra showed a decrease in intensity as indicated by the appearance of the isotropic peak coming from the contaminating deuterated water (Fig. 8D). This is probably due to the smearing out of the signal coming from the POPS molecules interacting with the protein. However enough POPS molecules remain free of interaction with protein to leave the onset of the transition at −4°C, i.e. the same than in the absence of protein.

**Figure 9:**
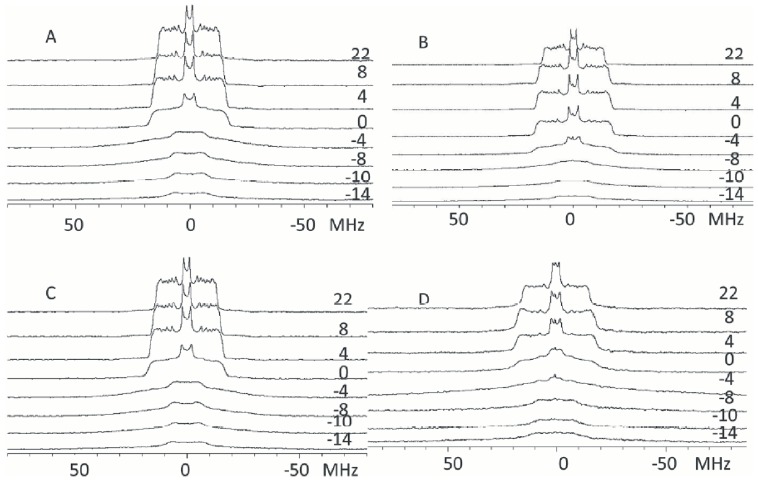
^2^H-NMR representative spectra obtained at the temperatures shown. (A) POPC/POPC-d_31_/POPS/PIP_2_ (35∶40∶20∶5, molar ratio); (B) POPC/POPC-d_31_/POPS/PIP_2_ (35∶40∶20∶5, molar ratio) in the presence of C2α (molar ratio phospholipid/protein 40∶1); (C) POPC/POPS-d_31_/PIP_2_ (75∶20∶5, molar ratio) and (D) POPC/POPS-d_31_/PIP_2_ (75∶20∶5, molar ratio) in the presence of C2 domain (40∶1 phospholipid/protein molar ratio).

When the mixture POPC/POPS-d_31_/PIP_2_ was studied (with a 75∶20∶5, molar ratio), the effect of adding PIP_2_ was insignificant since the pattern of the spectra (Fig. 9A) remain similar to those observed above for POPC/POPS mixtures (Fig. 8) in the absence of protein and also the transition temperature detected by plotting the *M_1_* values has again its onset at −4°C (Fig. 7C). The presence of C2α did not appreciably alter the transition behaviour of the system with respect to the same phospholipid mixture in the absence of protein and this can be said for both the spectra pattern (Fig. 8B) and the onset of the transition at −4°C as seen by the plot of the *M_1_* values (Fig. 7C). The addition of C2α domain to this last sample produced a decrease in the intensity of the spectra as it was also seen with POPC/POPSd_31_ as indicated by the appearance of the isotropic peak of the contaminant deuterated water. Nevertheless, the onset of the transition of the observed POPS molecules, remained at the same temperature, indicating that these spectra correspond to phospholipid which was not interacting with the protein.

A different result of adding C2α was observed when studying samples that included PIP_2_. The sample POPC/POPC-d_31_/POPS/PIP_2_, (35∶40∶20∶5, molar ratio) (Fig. 9A) shows a similar pattern of spectra than the sample without PIP_2_ (Fig. 7A). Also the onset of the transition was located at −4°C (Fig. 8E). However, although the spectra obtained after the addition of C2α domain to this membrane (Fig. 9B) were very similar to those obtained for pure phospholipid, the phase transition occurred at a lower temperature, for example at −4°C the pattern of the spectrum had already changed, showing that the transition had taken place at a lower temperature. As can be seen in Fig. 7C the phase transition studied by plotting *M_1_* values, was located at −8°C exactly as it happens for pure POPC (Fig. 7A) and in coincidence with the value observed before by using this same NMR technique [36]. This indicates that the association of the anionic phospholipid POPS with C2á demixed it from POPC when PIP_2_ was present. As the process leads to pure POPC, and as it was observed that PIP_2_ is also demixed by the protein domain (Fig. 5) it may be concluded that the PIP_2_ is also segregated.

## Discussion

We have studied the effect of adding C2α to a membrane system composed of POPC plus anionic phospholipids like POPS and PIP_2_, which are known to activate PKCα. We show using fluorescence quenching, that the C2α domain induces NBD-POPS demixing and that Ca^2+^ is indispensable for this effect. It is known that Ca^2+^ bridges the binding of the C2α domain to POPS [11], [14]. Additionally Ca^2+^ neutralizes negatively charged residues present in the Ca^2+^-binding site facilitating the approximation of the protein to the membrane where negatively charged are present [14], [37]. Thus, due to these reasons, it is explained why Ca^2+^ is so important, although the C2α domain also interacts through some amino acid residues directly with POPS [38]. Some proteins or peptides such as polylysine [39] are able to induce the capping of negatively charged phospholipids such as POPS, demixing them from amphipathic lipids, as POPC, and producing domains rich in polylysine and POPS and others poor in both. The aggregation of PIP_2_ by annexin A2 has been described recently [40].

The C2α domain is known to interact not only with phosphatidylserine through its calcium-binding site but it also binds one molecule of PIP_2_ with a high degree of affinity in another binding site located in a site rich in lysine residues [12], [18], [19]. Other phospholipids such as POPS and POPA [11] and also other molecules like retinoic acid [24] may also bind to this site but this site shows the highest affinity for PIP_2_ [12], [18], [19].

The very interesting observation, made in this work is that, in the absence of PIP_2_, little self-quenching was detected. However, in the presence of PIP_2_ the self-quenching of POPS-NBD was considerably higher. When TopFluor-PIP_2_ was used the C2α domain produced self-quenching but only when POPS is also present, indicating that the interaction of the domain with both POPS and PIP_2_ are necessary to produce anionic phospholipid demixing, although Ca^2+^ is also needed.

The site-directed mutagenesis of the C2α domain showed that both the Ca^2+^-binding site and the site rich in lysine residues are necessary for the self-quenching of POPS-NBD and the mutation of any of them almost abolish the lipid demixing. Therefore the fluorescence experiments indicate that demixing of anionic phospholipids is very much enhanced when both lipid binding sites of C2α are occupied, the Ca^2+^ binding site with this cation and phosphatidylserine and the site rich in lysine residues with PIP_2_. When POPC-NBD was used no self-quenching was detected, thus demonstrating that there is not a specific interaction of the NBD fluorophore moiety with the C2α domain and that the C2α domain establishes an electrostatic interaction with anionic lipids, which leads to their demixing and that an amphoteric lipid like POPC is not affected by this protein. When pure POPC was studied by ^2^H-NMR, C2α did not affect the phase transition, confirming that the domain does not interact with POPC. However it interacts with POPS as seen in the experiment in which it is added to pure phosphatidylserine, widening the phase transition.

When the samples containing POPC/POPSd_31_ and POPC/POPSd_31_/PIP_2_ were studied it was observed that the addition of the C2α domain reduced the intensity of the spectra as indicated by the isotropic peak produced by the contaminating deuterated water, due to the widening of the signal corresponding to POPS molecules interacting with the protein. The ^2^H-NMR experiments indicated that POPC is demixed with respect to the anionic lipids, and shows a phase transition at the same temperature than pure POPC-d_31_. This can be explained if most PIP_2_ molecules are also excluded from POPC and form part of a lipid domain rich in POPS and PIP_2_, where the C2α domain molecules are bound.

In the case of the sample with POPC/POPSd_31_/PIP_2_, it was observed that the presence of the C2α domain does not apparently affect the transition temperature of this phospholipid. We interpret this as competing influences on the transition temperature, since although the segregation of POPC should increase this temperature, PIP_2_ segregated with POPS should decrease it. The result is that no significant change was observed in the mixture deuterated POPS/POPC and PIP_2_ in the presence of C2α domain.

When POPCd_31_ and POPS are mixed, but in the absence of PIP_2_, the NMR experiments did not allow to observe any demixing, although the fluorescence experiments showed that this may happens although in a limited extent. This is probably due to the lower sensitivity of the NMR technique for this type of event.

We hypothesize that the reason why POPS demixing by the addition of C2α was greatly increased by the presence of PIP_2_ could be due to the great increase in the protein binding to the membrane that is observed when PIP_2_ is added to a POPC/POPS membrane [15], [18], [27] and to the different docking of the C2α protein in the presence of PIP_2_, which has been shown to change with respect to a membrane without this phosphoinositide, by both ATR-infrared spectroscopy [41] and EPR site-directed spin-labeling and relaxation [42].

The physiological significance of the observed anionic lipid demixing can be suggested, since the concentrations of phospholipids used in these experiments are close to the concentrations found in animal plasma membranes. It is known at this respect that the concentration of POPS in the inner monolayer of eukaryotic plasma membranes, such as in erythrocyte or platelet cells, is roughly 20 mol% [43]–[45]. The physiological concentration of PIP_2_ has been described to be around 1 mol% of the total lipid of plasma membranes [46], [47] and it is likely to be concentrated in the inner monolayer at 2 mol%, which increase locally if it forms clusters or patches [48]. Therefore, it is likely that demixing of POPS and PIP_2_ may take place in the plasma membrane induced by the presence of C2α domain.

It has recently been described that the C2 domains of synaptotagmin 1 are capable of segregating POPS within complex membranes [26], and changes have been detected in the physical properties of POPS molecules interacting with the C2 domains which probably lead to their demixing. Although there is a high homology between these domains, it seems that the C2 domains of synaptotagmin 1 do not need of the simultaneous presence of PIP_2_ to induce the demixing of anionic phospholipids; this can be explained, at least partially, by a recent demostration that shows that the C2A domain of sypnatotagmin 1 does not conserve a lysine residue critical for the interaction with PIP_2_ [4], [49], [50]. In the case of synaptotagmin C2AB or C2B, it was reported using ATR-IR that these domains disordered the lipid acyl chains of phosphatidylserine [26]. Our ^2^H-NMR results show (see Fig. 7B and 7C) that C2α disorders the acyl chains of POPS since *M_1_* values of POPC/POPS-d_31_ decreased in the presence of C2α at temperatures above the phase transition, when they are compared with the sample of just lipid. Nevertheless the decrease in the case of C2α is low and in some temperatures not significant.

In the case of synaptotagmin 1, the observed demixing may serve to facilitate membrane fusion. In the case of the C2α, the physiological implications are different and are probably linked to the activation of the enzyme, an effect supported by the establishment of domains rich in POPS and PIP_2_.
